# Anal cancer impact among people with HIV infection – a matched cohort study

**DOI:** 10.3332/ecancer.2025.1922

**Published:** 2025-06-03

**Authors:** Amanda Acioli de Almeida Robatto, Erika Andrade Rocha, Renata Colombo Bonadio, Denis Artico Galhera, Carolina Teixeira Muratori, Admir Andre Belo Bueno, Abraão Ferreira Lopes Dornellas, Luciana Bastos Valente Alban, Carolina Ribeiro Victor, Maria Ignez Freitas Melro Braghiroli, Marília Polo Mingueti e Silva, Camila Soares Araujo, Carlos Frederico Sparapan Marques, Caio Sergio Rizkallah Nahas, Karim Yaqub Ibrahim, André Tsin Chih Chen, Paulo Marcelo Gehm Hoff, Camila Motta Venchiarutti Moniz

**Affiliations:** 1 ICESP – Instituto do Câncer do Estado de São Paulo, Dr. Arnaldo Avenue, Sao Paulo 01246-000, Brazil; 2 IDOR – Instituto D’Or de Pesquisa e Ensino, Sao Paulo 01401-002, Brazil; 3 Universidade São Caetano do Sul, São Caetano do Sul 09521-160, Brazil; a https://orcid.org/0000-0002-0255-0765; b https://orcid.org/0009-0008-8275-0345; c https://orcid.org/0000-0001-5818-922X; d https://orcid.org/0000-0002-6534-3755; e https://orcid.org/0009-0007-1567-8665; f https://orcid.org/0009-0008-9140-2764; g https://orcid.org/0000-0003-3634-8503; h https://orcid.org/0009-0004-0147-8062; i https://orcid.org/0000-0001-8179-4639; j https://orcid.org/0000-0001-6366-8786; k https://orcid.org/0009-0002-9681-3374; l https://orcid.org/0000-0003-4675-3369; m https://orcid.org/0000-0003-4293-6301; n https://orcid.org/0000-0001-8036-512X; o https://orcid.org/0000-0002-5074-3860; p https://orcid.org/0000-0001-5183-1048; q https://orcid.org/0000-0002-0065-2194; r https://orcid.org/0000-0002-1182-4764

**Keywords:** anal carcinoma, squamous cell carcinoma of the anus, HIV, chemoradiation, CRT

## Abstract

**Background:**

Pivotal studies with curative chemoradiation (CRT) in anal cancer did not include HIV-positive (HIV+) patients. HIV status impact remains unknown in this scenario.

**Methods:**

In this retrospective matched cohort study, electronic medical records were reviewed at Sao Paulo State Cancer Institute between 2010 and 2021 patients with anal cancer T1-4 N0-1 M0 by AJCCVIII were selected. For each HIV+ patient, one or two HIV-negative (HIV-) cases were matched by age, stage (T, N) and ECOG. The primary endpoint was OS, estimated using Kaplan-Meir and compared with the log-rank test.

**Results:**

122 patients were selected, 45 being HIV+. The median follow-up was 37 months. Most patients, *n* = 119 (98%), received concomitant CRT and had ECOG 0/1 (*n* = 116, 95%). Stage III corresponded to 69% of the patients (*n* = 85). Positive nodes were detected in 76 patients (62%). No difference was observed in complete clinical response (cCR) post-CRT (68% in HIV+ versus 63% in HIV-; *p* = 0.6). Median recurrence-free survival (RFS) was not reached; 3-year RFS rates were 60.7% in HIV+ versus 63.1% in HIV- [hazard ratio (HR) 1.20, 95% CI 0.66–2.17, *p* = 0.538]. Median OS was not reached; 3-year OS was 66.4% HIV+ versus 72.2% in HIV- (HR 1.23, 95% CI 0.61–2.47, *p* = 0.546). HIV+ pts presented significantly more hospital admissions due to toxicity, 30% (*n* = 12/40) versus 13% (*n* = 10/74) (*p* = 0.049). No difference between groups was found for colostomy (*p* = 0.69) and salvage surgery (*p* = 1).

**Conclusion:**

Anal carcinoma HIV+ patients treated with CRT presented similar cCR, RFS and OS compared with HIV- patients. Optimal therapy should be attempted in the HIV+ population; however, close clinical monitoring due to higher hospital admission is required.

## Introduction

Anal cancer is a rare disease, accounting for just 0.5% of all new cancer cases [1]. Still, its age-adjusted death rate has been steadily rising, averaging an increase of 5.1% annually from 2013 to 2022 [1]. The 5-year relative survival rates for squamous cell carcinoma of the anus (SCCA) vary significantly depending on the disease stage: 84.5% for localised disease, 68.2% for regional disease and 36.3% for distant metastases [1]. SCCA is the most common subtype of anal cancer, representing approximately 90% of cases and is strongly associated with immunosuppression and human papillomavirus (HPV) infection [2].

People living with HIV/AIDS are at an increased risk for both *in situ* and invasive HPV-associated cancers, including anal cancer. The HIV population is 19 times more likely to develop anal cancer compared to the general population, with men at a higher risk (hazard ratio [HR], 20.73; 95% confidence interval [CI], 15.60–27.56) and women are also significantly at risk (HR, 12.88; 95% CI, 8.69–19.07) (3). Despite the high incidence of SCCA in HIV-positive (HIV+) patients, this population is often underrepresented in key prospective clinical trials on chemoradiotherapy (CRT) and the impact of CRT in this group remains poorly understood. This study aimed to evaluate differences in overall survival (OS), recurrence-free survival (RFS), complete clinical response (cCR), toxicity, colostomy and salvage surgery between HIV+ and HIV-negative (HIV-) patients with localised SCCA undergoing standard-of-care curative treatment.

## Materials and Methods

This is a retrospective matched cohort study. We reviewed electronic medical records from 2010 to 2021 at the Instituto do Câncer do Estado de São Paulo, Brazil.

### Ethical consideration

This study followed the guidelines of the Declaration of Helsinki. It was approved by the ethics committee and the institutional review board of Instituto do Câncer do Estado de São Paulo, Brazil. Due to the retrospective nature of the study, the requirement for informed consent was waived.

### Patients

Inclusion criteria were histologically confirmed invasive SCCA and stage I-III by AJCC VIII Edition treated with curative intent.

### Endpoints

The primary endpoint was OS. The secondary endpoints were RFS, cCR, toxicities, colostomy and salvage surgery.

### Statistical analysis

HIV+ patients were matched using the R package ‘MatchIt’ with one or two non-metastatic HIV- cases, based on age, stage (T, N) and ECOG performance status, as these were considered the primary matching criteria.

The OS, RFS and cCR were estimated using Kaplan-Meir and log-rank tests. Toxicities were evaluated in patients who received CRT. To handle missing data in toxicities analyses, we excluded patients with no information about specific variables. A maximum of five patients were excluded from each group, as detailed in the supplementary material (Supplemental Tables 1 and 2). The *p*-value for toxicities during CRT was evaluated using the chi-square test.

The comparison between HIV+ and HIV- patients for colostomy and salvage surgery were analysed using Fisher’s exact test.

## Results

We included 122 patients, 45 HIV+ patients, with a median follow-up of 37 months. The median age was 59 years in the HIV- group and 48 in the HIV+ group. We observed a high prevalence of males in the HIV+ group patients (*n* = 31, 68.8%) and females (*n* = 62, 80.5%) in the HIV- group. Stage III was the most frequent in both groups; most T4 (*n* = 41, 33%) and T3 tumours (*n* = 36, 29%). Positive nodes were detected in *n* = 76 (62%) patients (Table 1). Most patients (*n* = 119, 98%) received concomitant CRT as treatment with curative intent and had ECOG 0/1 (*n* = 116, 95%). Definitive concomitant regimens included mitomycin C (MMC) plus infusional 5-Fluorouracil (5-FU) (*n* = 37; 31%), cisplatin (CDDP) plus capecitabine (*n* = 26; 21,8%) and CDDP plus infusional 5-FU (*n* = 35; 29%). Patients received a median of 54 Gy in 30 fractions in the primary tumour and 45 Gy in elective lymph node drainage. Three-dimensional conformal radiotherapy, intensity-modulated radiotherapy (IMRT) and volumetric-modulated arc therapy (VMAT) were used in 23%, 47% and 29% of patients, respectively.

**Figure 1.**
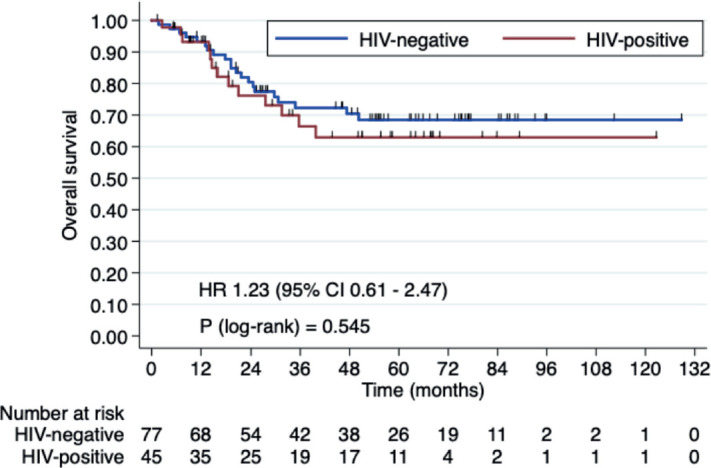
OS in HIV+ patients versus HIV- patients.

**Figure 2.**
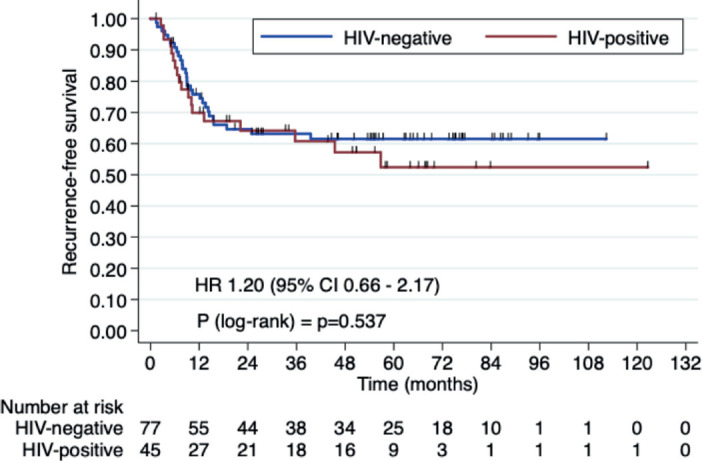
RFS in HIV+ patients versus HIV- patients.

Three-year OS rates were 66.4% in HIV+ patients versus 72.2% in HIV- patients (HR 1.23, 95% CI 0.61–2.47, *p* = 0.546). Three-year RFS rates were 60.7% in the HIV+ group versus 63.1% HIV- group (HR 1.20, 95% CI 0.66–2.17, *p* = 0.538). Median OS and RFS were not reached (Figures 1 and 2).

cCR at 6 months post-CRT was 68% in HIV+ patients and 63% in HIV- patients (*p* = 0.6) (Table 2).

HIV+ patients had significantly more hospital admissions due to toxicity (*n* = 12, 30%) than HIV- (*n* = 10, 13.8%) (*p* = 0.049) (Table 3). The main toxicities were radiodermatitis in both groups, 45% in HIV+ patients and 40% in HIV- patients and lymphopenia, 50% in HIV+ patients and 43% in HIV- patients (Table 4).

Some patients required a colostomy or salvage surgery before, during or immediately after CRT. When analyzing only the patients who underwent CRT, colostomy was performed in 29.5% of HIV+ patients and 33.3% of HIV- patients (Table 5). Salvage surgery was performed in 11.3% of HIV+ patients and 10.6% of HIV- patients. No statistically significant differences were found when comparing patients based on HIV status. The odds ratio (OR) for colostomy was 0.84 (95% CI, 0.34–2.0; *p* = 0.69), and for salvage surgery, it was 1.05 (95% CI, 0.25–3.97; *p* = 1).

## Discussion

SCCA is a rare cancer, representing just 0.5% of all new cancer cases, according to the SEER database. Early diagnosis and timely treatment are crucial for better outcomes since 84.5% of patients with localised disease and 68.2% with regional disease are alive in 5 years [1]. Preventable risk factors are associated with SCCA, with HPV being the most prominent, particularly when combined with HIV infection [3]. As a result, offering HIV testing to patients newly diagnosed with SCCA is strongly recommended [4].

The standard treatment for non-metastatic SCCA involves CRT with a fluoropyrimidine, such as 5-fluorouracil and an MMC regimen, which yields a 67% OS rate at 5 years [5]. An alternative treatment option is CDDP combined with fluoropyrimidine, with no significant differences in complete response rates or progression-free survival (PFS) between the CDDP and MMC groups [6]. However, HIV+ patients were not included in these phase III clinical trials, so data on key endpoints such as OS, PFS and toxicity in this population remains unavailable.

Contemporary studies are increasingly focusing on the HIV+ population. In a prospective cohort study that included 12% HIV+ patients treated with a full dose of MMC and fluoropyrimidine, HIV positivity was associated with a worse response rate (OR 5.72; 95% CI 2.5–13.0; *p* < 0.001). The 5-year PFS and OS rates were 63.3% and 76.4%, respectively, with a median follow-up of 66 months. Multivariate analysis revealed that the absence of complete response at 6 months (HR 3.36, *p* = 0.007, 95% CI 1.39–8.09) was associated with inferior OS [7]. In a Phase II prospective study evaluating MMC plus capecitabine during CRT, 9.3% of the participants were HIV+, with an OS of 97.7% at a median follow-up of 23.1 months; however, there was no comparison between HIV+ and HIV- patients [8].

In our current study, we aimed to assess whether there is a significant difference in outcomes between HIV+ and HIV- patients undergoing CRT. Our findings showed that the 3-year OS rates were 66.4% for HIV+ patients versus 72.2% for HIV- patients (HR 1.23, 95% CI 0.61–2.47, *p* = 0.546). The 3-year RFS rates were 60.7% for the HIV+ group and 63.1% for the HIV- group (HR 1.20, 95% CI 0.66–2.17, *p* = 0.538). Median OS and RFS were not reached. These results support the idea that HIV+ status does not significantly affect CRT outcomes (OS and RFS) when properly managed.

Another important consideration is that HIV+ patients in the era of modern antiretroviral therapy can achieve adequate viral load suppression and maintain healthy CD4 counts. However, due to the absence of established guidelines and limited recent data on CRT tolerance and outcomes in the HIV+ population, adjustments to radiotherapy and chemotherapy doses are still made at the discretion of the treating physician.

Regarding toxicities, three retrospective studies involving HIV-infected patients during CRT reported relevant data. One of the most common toxicities was grades 3 and 4 dermatitis, which ranged from 23% to 50%. Grades 3 and 4 hematological and gastrointestinal toxicities were also frequently observed, with rates ranging from 30% to 38% and 15% to 30%, respectively [9, 10, 11]. Additionally, neutropenia and lymphopenia were commonly observed in HIV+ patients undergoing CRT [12]. A small retrospective cohort also identified a pretreatment CD4 count below 200 as a factor associated with poorer anal cancer control and increased treatment-related morbidity [13]. In this cohort, HIV+ patients more frequently experienced lymphopenia (50%) and radiodermatitis (45%) as grade 3 and 4 toxicities. Furthermore, they had significantly higher rates of hospitalisation due to toxicity (30%, *n* = 12/40) compared to HIV- patients (13.8%, *n* = 10/72; *p* = 0.049).

Quality of life (QoL) is another crucial outcome for patients with anal cancer, particularly for those who are HIV+. Several factors influence their QoL, including the impact of a colostomy, cancer treatment and HIV itself. Treatment for anal cancer, typically involving chemoradiotherapy, can lead to significant side effects such as bowel dysfunction, fatigue and sexual dysfunction, which may persist long-term and significantly affect QoL [14, 15, 16]. The addition of a colostomy further complicates these issues, often resulting in body image concerns, challenges with social functioning and sexual health difficulties [17]. These physical and psychosocial burdens can lead to a marked decline in overall QoL. Although studies specifically focusing on HIV+ patients with anal cancer and colostomies are limited, related research indicates that their QoL may be further compromised compared to HIV- patients. The existing literature underscores the need for more targeted research to better understand the unique QoL outcomes in this population and address both the physical and psychosocial factors critical to improving their overall well-being [17, 18].

As a retrospective cohort study, our data regarding QoL were limited; however, the use of colostomy and salvage surgery was evaluated. Among HIV- patients who underwent CRT, 33.3% required a colostomy and 10.6% underwent salvage surgery. Among HIV+ patients, 29.5% required a colostomy and 11.3% underwent salvage surgery. No statistically significant difference was found when comparing HIV+ to HIV- groups: the OR for colostomy was 0.84 (95% CI 0.34–2.0; *p* = 0.69) and for salvage surgery was 1.05 (95% CI 0.25–3.97; *p* = 1).

Our study’s retrospective design has inherent limitations, including small sample size, missing data due to variability in patient registries, lack of patient stratification by CD4 count and specific CRT regimens. Patients were not stratified by CD4 count due to its unavailability for all subjects. Additionally, stratification by treatment regimen was not performed, as we did not anticipate any imbalance between the groups.

However, since most patients received standard CRT, our findings offer valuable insights into the safety and efficacy of CRT in HIV+ individuals. Our data suggest that HIV+ patients with good performance status (ECOG 0–1) can safely undergo standard CRT. However, due to their increased risk of toxicity, vigilant monitoring and timely treatment adjustments are crucial. Efforts are underway to include the HIV+ population in prospective studies, such as the Phase III POD1UM-303/InterAACT 2 trial [19]. However, this study focused on metastatic SCCA. Future prospective studies involving patients undergoing CRT for localised SCCA should aim to identify optimal treatment strategies for this population, incorporating immunological parameters like CD4 count and HIV viral load to refine treatment guidelines.

## Conclusion

In this matched cohort study, HIV+ patients with localised anal carcinoma and good performance status who were treated with standard CRT showed similar outcomes in terms of OS, RFS, cCR, colostomy and salvage surgery compared to HIV- patients. Therefore, optimal therapy should be pursued for the HIV+ population, with close clinical monitoring due to the significantly higher risk of hospital admissions related to treatment toxicity in this group.

It is crucial to include HIV+ patients in ongoing prospective clinical trials, particularly those involving novel targeted therapies with expected lower bone marrow toxicity, to provide future evidence on response rates and treatment tolerance within this population.

## Conflicts of interest and funding declaration

The authors declare no conflicts of interest related to the development of this work. This research did not receive any specific grant from funding agencies in the public, commercial or not-for-profit sectors.

## Prior presentation

Presented as Poster at European Society of Medical Oncology (ESMO) Congress 2022, September 9–14, 2022.

## Figures and Tables

**Table 1.** Characteristics of the population.

	HIV+, *n* (%) *n* = 45	HIV -, *n* (%) *n* = 77	Total, *n*
Gender			
Female	14 (31)	62 (80.5)	76
Male	31 (68.8)	15 (19)	46
Age (median)	48 (27–69)	59 (38–80)	
Type			
Squamous cell carcinoma	45 (100)	77 (100)	122
HPV			
Positive	5 (11)	16 (20.7)	21
Negative	2 (4)	6 (7.9)	8
Unknown	38 (84.4)	55 (71)	93
Staging AJCC VIII			
I	1 (2)	2 (2.5)	3
IIA	6 (13)	12 (15.5)	18
IIB	7 (15.5)	9 (11.6)	16
IIIA	9 (20)	14 (18)	23
IIIB	3 (6.6)	7 (9)	10
IIIC	19 (42)	33 (42.8)	52
T staging			
T1	2 (4)	3 (3.8)	5
T2	14 (31)	26 (33.7)	40
T3	15 (33)	21 (27)	36
T4	14 (31)	27 (35)	41
N staging			
N0	17 (37.7)	29 (37.6)	46
N1a	12 (26.6)	23 (29.8)	35
N1b	3 (6.6)	4 (5)	7
N1c	13 (28.8)	21 (27)	34
ECOG			
0	19 (42)	27 (35)	46
1	23 (51)	47 (61)	70
2/3	3 (6.6)	3 (3.8)	6
Treatment modality			
RT alone	1 (2)	1 (1)	2
CRT	44 (97.7)	75 (97)	119
Other	0	1 (1)	1
Types of concomitant CT			
MMC + Capecitabine	2 (4)	13 (16.8)	15
MMC + 5-FU infusional	13 (28.8)	24 (31)	37
CDDP + Capecitabine	11 (24)	15 (19)	26
CDDP + 5-FU infusional	13 (28.8)	22 (28.5)	35
5-FU infusional	4 (8)	1 (1)	5
Capecitabine	1 (2)	0	1
RT modality			
3D	10 (22)	15 (19)	25
IMRT	17 (37.7)	35 (45)	52
VMAT	12 (26.6)	20 (25.9)	32
Other	1 (2)	1 (1)	2
Unknown	5 (11)	6 (7.7)	11

RT = radiotherapy; CRT = chemoradiotherapy; CT = chemotherapy;

MMC = Mitomycin; 5-FU = 5-fluorouracil; CDDP = Cisplatin

**Table 2.** Outcomes of CRT in HIV+ and HIV- patients.

	HIV+ (%)	HIV- (%)	*p-value*
cCR	68	63	0.6
3y-RFS	60.7	63.1	0.53
3y-OS	66.4	72.2	0.54

cCR = complete clinical response; RFS = recurrence free survival;

OS = overall survival

**Table 3.** Toxicities during CRT.

	HIV+, n (%) *n* = 44*	HIV-, n (%) *n* = 75**	*p-value*
CT dose reduction due to toxicity	3 (7)	8 (10.6)	0.531
CT suspension due to toxicity	6 (13.9)	13 (17.5)	0.362
Pause of RT during treatment	13 (29.5)	22 (30)	0.922
Treatment suspension due to toxicity	8 (19)	17 (23)	0.578
Hospitalization during treatment	**12 (30)**	10 (13.8)	**0.049**

CT = chemotherapy; RT = radiotherapy

* One patient was excluded due to no CRT treatment

** Two patients were excluded due to no CRT treatment

Statistical significance at *p* < 0.05

**Table 4.** Adverse events of CRT.

Toxicity (G3/G4)	HIV+, *n* (%) *n* = 44*	HIV-, *n* (%) *n* = 75**	*p-value*
Radiodermatitis	20 (45)	29 (40)	0.613
Nausea	0	5 (6.8)	0.075
Vomiting	0	4 (5)	0.116
Diarrhea	3 (7)	4 (5)	0.730
Vasospasm	1 (2)	1 (1)	0.690
Anemia	3 (7.5)	4 (5.5)	0.695
Neutropenia	5 (12.5)	4 (5.5)	0.219
Febrile neutropenia	2 (4.8)	2 (2.6)	0.571
Lymphopenia	20 (50)	31 (43)	0.600
Thrombocytopenia	4 (10)	1 (1)	0.032
Any toxicities G3/4	26 (59)	49 (66)	0.408

* One patient was excluded due to no CRT treatment

** Two patients were excluded due to no CRT treatment

**Table 5.** Colostomy and salvage surgery HIV+ versus HIV- patients.

	HIV+ (%) *n* = 44*	HIV- (%) *n* = 75**
Colostomy	13 (29.5%)	25 (33.3%)
Salvage surgery	5 (11.3%)	8 (10.6%)

* One patient excluded due to no CRT treatment

** Two patients excluded due to no CRT treatment

## Supplementry

**Supplemental Table 1.** Excluded patients from analysis of toxicities during CRT per group due to missing data.

	HIV+ *n* = 44*	HIV- *n* = 75**
CT reduction due to toxicity	2	0
CT suspension due to toxicity	1	4
Pause of RT during treatment	0	2
Treatment suspension due to toxicity	2	2
Hospitalization during treatment	**4**	3

* One patient excluded due to no CRT treatment

** Two patients excluded due to no CRT treatment

Statistical significance at *p* < 0.05

**Supplemental Table 2.** Excluded patients from analysis of adverse events of CRT per group due to missing data.

Toxicity (G3/G4)	HIV+ *n* = 44*	HIV- *n* = 75**
Radiodermatitis	0	3
Nausea	0	2
Vomiting	2	3
Diarrhea	2	2
Vasospasm	2	3
Anemia	4	4
Neutropenia	4	5
Febrile neutropenia	3	4
Lymphopenia	3	4
Thrombocytopenia	5	4
Any toxicities G3/4	0	1

* One patient excluded due to no CRT treatment

** Two patients excluded due to no CRT treatment
